# From Goals to Gains: Therapy Goal Selection, Attainment, and Their Association With Psychotherapy Outcomes

**DOI:** 10.32872/cpe.19567

**Published:** 2026-08-31

**Authors:** Flavio Iovoli, Saskia Rieger, Juan Martín Gómez Penedo, Jessica Fritz, Martin Cordes, Ruben Lauterbach, Henning Schöttke, Julian A. Rubel

**Affiliations:** 1 Department of Psychology, Osnabrueck University, Osnabrueck, Germany; 2 Department of Psychology, University of Kassel, Kassel, Germany; 3 Department of Psychology, Vrije Universiteit Brussel, Brussels, Belgium; 4 Department of Medicine, Justus-Liebig-University Giessen, Giessen, Germany; Friedrich-Alexander-Universität Erlangen-Nürnberg, Erlangen, Germany

**Keywords:** therapy goals, goal selection, goal attainment, psychotherapy outcomes, individualized outcomes

## Abstract

**Background:**

Therapy goals are central to psychotherapeutic interventions. While symptom-specific goals are commonly emphasized, patients often set diverse goals that may not align with standardized symptom measures. This study examines how patient characteristics are associated with the selection of specific therapy goal content categories and whether attainment in these domains shows associations with different psychotherapy outcomes.

**Method:**

Therapy goals from 645 patients undergoing cognitive-behavioral therapy in an outpatient clinic were categorized using the Bern Inventory of Treatment Goals and analyzed. Baseline predictors of therapy goal selection and diversity were examined using multilevel logistic and ordinal regression models. The association between goal selection and their goal attainment with residualized post-treatment scores in symptoms, global functioning, and end-of-treatment patient satisfaction were assessed using Bayesian multilevel regression models.

**Results:**

Baseline patient characteristics were associated with the selection of specific therapy goal categories, alongside substantial therapist-level effects in several categories. Goal-category attainment showed differential associations with psychotherapy outcomes, with the strongest and most consistent effects observed for symptom-related outcomes.

**Conclusion:**

These findings indicate that the attainment of specific therapy goal categories is meaningfully reflected in standardized outcome measures, but in a category-sensitive rather than uniform way. Combining therapy goal-based and standardized assessments may improve the alignment between patient priorities and outcome evaluation in routine psychotherapy.

## Highlights

- Patient characteristics are associated with therapy goal selection and diversity.
- Goal attainment predicts global outcomes and represents valuable individualized indicators.
- Therapist effects are present in therapy goal selection and treatment results.

Therapy goals are a core element of many psychotherapeutic treatments (Grawe, 2004; Grosse Holtforth et al., 2004). They are individualized and an intentional expression of the changes a patient hopes to achieve through psychotherapy (Brunstein et al., 1998; Grosse Holtforth & Grawe, 2000). Naturally, patients seek psychotherapy for a variety of reasons (Michalak et al., 2004). Some may want to gain control over their thoughts, while others aim to overcome specific fears or enhance their overall sense of well-being. Some individuals seek therapy to learn relaxation techniques or improve self-control. Others might be searching for deeper meaning and purpose in their lives. Given these diverse objectives, therapy goals shape the therapeutic process (Grosse Holtforth et al., 2004; Michalak & Schulte, 2002; Silberschatz, 2017). In particular, collaboratively defined therapy goals are associated with patient engagement and motivation (Grosse Holtforth & Michalak, 2012; Michalak & Grosse Holtforth, 2006) and represent a core component of the therapeutic alliance (Bordin, 1979; Tryon, 2018; Tryon & Winograd, 2011).

However, as illustrated above, patients’ individual therapy goals might not always align with disorder-specific symptoms (Chevance et al., 2020; Lloyd et al., 2019; Ramnerö & Jansson, 2016b). Despite this, psychotherapy effectiveness is often assessed through changes in standardized symptom checklists (for example, the SCL-90-R; Derogatis, 1977), which may not fully represent the patient’s focus in psychotherapy. This approach risks overlooking the very important aspect of psychotherapy success: achieving the goals that patients sought psychotherapy for and consider most relevant. In fact, a meta-analysis by Lindhiem and colleagues (2016) demonstrated that psychotherapy shows significantly greater effectiveness when outcomes are measured through idiographic, goal-specific outcomes rather than using standardized symptom checklists. Although symptom-specific measures are convenient and provide opportunities for nomothetic comparison (Lambert et al., 2005), they are less consistently implemented in routine care than in research contexts (Ionita & Fitzpatrick, 2014; Puschner et al., 2015). Therapists sometimes perceive these measures as time-consuming, potentially burdensome for some patients, and ultimately not useful in practice (Gleacher et al., 2016; Hatfield & Ogles, 2007). In line with this, Jensen-Doss and colleagues (2018) found that therapists in practice tend to use and prefer idiographic outcome measures over standardized symptom-specific ones. Together, these highlight the importance of systematically examining patient-defined therapy goals and their relationship to established outcome indicators.

The most widely used assessment method for capturing idiographic therapy goals and their progress (Lloyd et al., 2019; Sales, Ashworth, et al., 2023) is the Goal Attainment Scaling (GAS; Kiresuk et al., 1994; Kiresuk & Sherman, 1968). In GAS, patients and therapists jointly define up to five individualized goals and specify criteria for different levels of attainment, which are then evaluated during or at the end of treatment (Kiresuk et al., 1994). While GAS provides a structured way to assess goal attainment, its highly individualized format limits comparability across patients. To address this limitation, the Bern Inventory of Treatment Goals (BIT) offers an empirically derived framework categorizing therapy goals by content (Grosse Holtforth, 2001; Grosse Holtforth & Grawe, 2002). Based on large samples of patient-reported goals, the BIT distinguishes five overarching themes (Grosse Holtforth & Grawe, 2002): (1) symptom- and problem-related (e.g., to overcome ruminative thoughts or learn to manage anxiety), (2) interpersonal-related (e.g., to improve relationships with specific individuals in personal or professional life), (3) personal growth-related (e.g., to develop greater self-confidence and self-assurance), (4) existential-related (e.g., to gain greater clarity about who I am, what I can do, and what I want), and (5) well-being-related (e.g., to make my leisure time more active; Grosse Holtforth, 2001) goals. Combining structured goal attainment ratings with standardized goal content categories enables systematic and comparable analyses of which types of goals patients pursue and how their attainment relates to broader treatment outcomes (Michalak et al., 2007). However, research in this area remains limited and fragmented, often focusing either on goal selection or on goal attainment alone, while little is known about how specific categories of therapy goals relate to treatment outcomes, despite the clear relevance of such knowledge for clinical practice.

Prior research on therapy goal selection suggests that patients often choose symptom- and problem-focused goals (Baur et al., 2024; Berking et al., 2005; Ramnerö & Jansson, 2016a; Sawrikar et al., 2023; Schöttke et al., 2014), whereas other work suggests a stronger emphasis on interpersonal or personal growth–related goals (Grosse Holtforth & Grawe, 2002). These differences likely also reflect contextual factors such as therapeutic orientation and treatment setting, which are associated with goal formulation (Dirmaier et al., 2006; Schöttke et al., 2014). Some variability appears to be associated with diagnostic differences, with anxious patients tending to select more symptom-focused goals and depressed patients showing more thematically diverse goal profiles, indicating differences not only in goal type but also in overall goal diversity (Grosse Holtforth et al., 2009). In this context, goal diversity refers to the extent to which a patient’s therapy goals span multiple thematic goal categories rather than concentrating on a single domain. Another study found that patient age was associated with differences in goal focus, with older patients emphasizing well-being and younger patients emphasizing personal growth (Sittler et al., 2022). However, the association between other baseline patient characteristics and both therapy goal selection and goal diversity remains largely unexplored. Clarifying these associations could support more tailored treatment planning and improve the alignment between therapeutic interventions and patient priorities.

While understanding which goals are selected seems important, an equally central question concerns whether and how the attainment of different therapy goals relates to treatment outcomes. Whether therapy goals are attained is clinically meaningful because goal attainment reflects tangible therapeutic progress from the patient’s perspective. Prior research indicates that attainment rates vary across goal categories, yet findings are mixed and show no consistent pattern, with some studies reporting higher attainment for well-being (Berking et al., 2005) or existential goals (Baur et al., 2024) and others for symptom-focused goals (Trachsel et al., 2008). These descriptive differences indicate that goal types are not equally easy to attain, yet they do not clarify whether attaining certain kinds of goals is more strongly linked to therapy outcome. Research connecting goal attainment to outcomes has mostly relied on overall attainment across all goals, which is associated with improvements in quality of life and reductions in anxiety and depression, and shows moderate correlations with standardized symptom change (Baur et al., 2024; Ramnerö & Jansson, 2016b). Category-specific predictive analyses are less common but particularly informative. A notable example is Schöttke et al. (2014), who analyzed a mixed sample of CBT and psychodynamic outpatients and tested whether the number of patient-defined goals within specific goal categories predicted treatment outcome. They showed that including patient-defined goal categories improved the prediction of outcome beyond standardized measures alone, and that having more symptom-focused goals was associated with poorer treatment success. However, their study focused on which goals were selected and how many goals fell into each category, not on whether these goals were actually attained. Thus, it remains largely unclear whether the attainment of specific types of therapy goals (beyond their selection) differentially relates to treatment outcomes. 

The present study addresses these gaps with two main objectives:

1. It examines how baseline patient characteristics are associated with therapy goal selection, specifically the likelihood of selecting each goal category at least once and the diversity of selected goals.
2. It investigates how both the selection and the attainment of specific goal categories relate to psychotherapy outcomes. Extending prior work that focused primarily on goal selection (Schöttke et al., 2014) or overall attainment (Baur et al., 2024; Ramnerö & Jansson, 2016b), this study tests whether category-specific goal attainment differentially predicts multiple outcome domains, ranging from symptom change to broader indicators such as global functioning and patient satisfaction.

Together, these analyses aim to clarify how individualized therapy goals connect to standardized outcome measures and to inform more patient-tailored treatment planning.

## Method

### Sample

The data were drawn from an archival outpatient psychotherapy dataset comprising 1,116 patients. For the present study, patients were included if they had reported at least three therapy goals. The resulting analytic sample consisted of 645 patients. The patients were on average 36.88 years old (*SD* = 13.95), almost two-thirds identified as female (62.6%), and reported to be in a relationship (64.9%). The majority of patients (58.9%) were primarily diagnosed with an affective disorder (ICD-10 F3; World Health Organization, 2004), most commonly recurrent major depressive disorder (moderate episode; 35.8%) and a single episode of major depressive disorder (moderate; 29.1%). Besides depressive disorders, prevalent diagnoses in the present sample were anxiety disorders (20.2%), reactions to severe stress and adjustment disorder (10.5%), somatoform disorder (6.7%), personality disorder (5.5%), and eating disorder (3.9%). Half of the patients (51.6%) were diagnosed with at least one comorbid disorder, where the mean number of diagnoses was 1.69 (*SD* = 0.83, range = 1-7).

### Treatment

The treatment was an evidence-based cognitive-behavioral therapy (Margraf & Schneider, 2009) in the outpatient clinic of Osnabruck University in Northwest Germany. Treatments were conducted by master-level psychologists in advanced clinical training and were regularly supervised by experienced licensed psychotherapists. All trainees had previously obtained their master’s degree in psychology and at least two years of postgraduate training. There were 105 therapists (77.8% female) in the sample, each treating an average of 6.14 patients (*SD* = 3.16). The number of patients treated by individual therapists ranged from 1 to 13.

### Measurements

#### Therapy Goals and Their Attainment

Therapy goals were categorized using the five main categories of the Bern Inventory of Treatment Goals – Checklist (BIT-C; Grosse Holtforth, 2001): symptom- and problem-related, interpersonal-related, personal growth-related, existential-related, and well-being-related goals. Only these five main categories were used in the present analyses. Each goal in the BIT-C is pre-assigned to one main category via its item code, and these predefined assignments were used directly in the dataset. The patients select their so-called naive therapy goals, which are then specified together with the therapist, before the start of the treatment (Grosse Holtforth, 2001). The formulated goals are the basis for the GAS (Kiresuk et al., 1994). Goal attainment is finally assessed by both the patient and therapist at the end of therapy. The GAS is rated on a 7-point rating scale (-3 "very far from achieved" to +3 "fully achieved") and shows good reliability, depending on the conditions of its application (e.g., Krasny-Pacini et al., 2016).

#### Symptom Distress

General psychological distress was assessed using the German version of the Symptom Checklist - Revised (SCL-90-R; Franke, 2002). The SCL-90-R is a self-report inventory consisting of 90 items, which evaluates psychological symptoms in the last seven days. Items are rated on a 5-point Likert scale ranging from 0 (not at all) to 4 (extremely). The mean score of the SCL-90-R is referred to as the global severity index (GSI) and indicates the general psychological distress of an individual. The patients in the current study completed the SCL-90-R before (t1) and after (t2) treatment. The instrument had excellent reliability (Cronbach’s α_t1_ = .97 and α_t2_ = .97).

#### Global Functioning

The German version of the Outcome Questionnaire-30 (OQ-30; Lambert et al., 2002) was used to evaluate the psychotherapy outcome as a more global measure than just symptom distress. The OQ-30 measures the functioning level of the past seven days with 30 items, which patients rate on a 5-point Likert scale from 0 (never) to 4 (almost always). The global functioning is assessed across three areas: symptom distress, interpersonal relationships, and social integration. In previous studies, the instrument has shown good reliability (Cronbach’s α = .93) and test-retest-reliability (α = .88; Lambert et al., 2002). In the current study, the OQ-30 showed good reliability (Cronbach’s α_t1_ = .80 and α_t2_ = .85).

#### Patients’ Treatment Satisfaction

Patient satisfaction was assessed at the end of the treatment, using the German version of the Client Satisfaction Questionnaire (CSQ-8; Schmidt et al., 1989). The CSQ-8 is a self-report questionnaire, on which patients rate their satisfaction with the treatment on 8 items with a 4-point Likert scale (1 = lowest; 4 = highest). Previously, the instrument has shown excellent reliability (Cronbach’s α = .92; Schmidt et al., 1989). In the current study, the reliability was good, Cronbach’s α = .86, at the end of the treatment.

### Procedure

The data were part of the routine outcome monitoring assessment of the outpatient clinic. Patients provided written informed consent and completed the baseline questionnaires. During the probationary treatment period (consisting of the first six to eight sessions), patients and therapists collaboratively selected a maximum of five therapy goals (consistent with the standard GAS framework) using the BIT-C. The goal number of the BIT-C allowed for the goals to be categorized into one of the five categories described above. These therapy goals were then specified by the patients and therapists, and if needed, refined in a person-specific way. This formulation was used as the basis for the goal attainment rating at the end of the treatment. For the purpose of the current study, a minimum number of goals was required to ensure sufficient data for analyzing how different goal categories impact therapy outcomes (original sample size = 1,116). Therefore, patients were included when they had at least three goals provided, and when their attainment was rated at the end of the psychotherapeutic intervention (*n* = 645).

### Data Analysis

#### Predicting Goal Selection

In order to investigate the factors predicting the selection of therapy goals across the five predefined goal-categories (symptom-focused, interpersonal, personal-growth, existential, and well-being goals), we employed a series of multilevel logistic regression models (Wong & Mason, 1985). These models were implemented using the *lme4* package (Bates et al., 2015). Each model treated the binary indicator of goal selection (0 = not selected, 1 = selected at least once) as the outcome variable and included the following baseline predictors: age, gender, baseline symptom severity as measured by the SCL-90-R global severity index and its subscales (*z*-standardized), and global functioning as measured by the mean of the OQ-30 and its subscales (*z*-standardized). A random intercept for therapists was incorporated to account for the nested data structure, where patients were grouped within therapists. To investigate the factors predicting goal diversity, defined as the number of different therapy goal categories selected by a patient (range: 1-5), we employed multilevel ordinal regression using the *clmm* function from the *ordinal* package (Christensen, 2023).

#### Predicting Treatment Outcome by Goal Selection and Attainment

To create the predictor variables, we calculated each patient’s mean attainment score for each goal category. Specifically, if a patient selected more than one goal from a category, we averaged the scores for those goals; if a patient did not select any goal from a category, the cell remained empty (“*NA”*) to indicate non-selection. In addition, a separate binary indicator was created to show whether a category was selected at least once.

Consequently, the predictors included the following two parameters for the five goal categories (symptom-related, interpersonal-related, personal growth-related, existential-related, and well-being-related):

- the goal selection: 1 = yes, the goal was selected at least once; 0 = no, the goal was never selected
- the patient’s mean attainment score (conditional on selection): a score ranging from -3 (much less attained than expected) to 3 (much more attained than expected), with NA indicating that the category was never chosen by the patient, and being modeled as structurally missing, with attainment effects estimated only for selected categories (selection × attainment interaction).

Outcome variables were defined as residualized post-treatment scores for symptom distress and psychotherapy outcome (PO; Cronbach & Furby, 1970). The post-treatment scores were regressed on the pre-treatment scores,


$$Predicted\;Post_{PO}=\;\beta_{0}+\;\beta_{1}{\text{x Pre}}_{PO\;}$$

and the difference between the observed and the predicted post-treatment score was computed,


$$Residualized\;Post_{PO}={\text{ Observed Post}}_{PO}\;-{\text{ Predicted Post}}_{PO\;}$$

where lower residualized scores indicate lower symptom distress than expected based on baseline severity.

Since goal attainment scores were only available for selected goal categories, missing values in the goal attainment predictors indicated structural non-applicability (meaning that a specific goal was never selected) rather than random missingness. In standard regression models, such missing predictor values would typically lead to listwise deletion of cases. To avoid this information loss, Bayesian multilevel regression models were estimated using the *brms* package (Bürkner, 2017), applying joint modeling of predictors with structurally missing values via the *mi*() specification. For each attainment predictor, an intercept-only auxiliary submodel (*mi*() ~ 1) was included. Attainment effects were modeled conditionally on goal selection using interaction terms (selection × attainment), so that attainment contributed to the outcome model only for categories that were actually selected, and cases were not additionally lost due to missing attainment predictors. Separate models were estimated for residualized posttreatment symptom distress, residualized posttreatment global outcome, and post-treatment patient satisfaction.

Random intercepts for therapists accounted for clustering within therapists. The intraclass coefficient (ICC) was calculated, which indicates the proportion of total variance attributable to the therapist-level in percent (Bryk & Raudenbush, 1987; Devine et al., 2024; Raudenbush & Bryk, 2002). The data analyses were performed in the R environment (Version 4.4.2, R Core Team, 2018).

## Results

### Descriptives

Table 1 presents descriptive statistics for goal selection and goal attainment across categories. The ranking of selected therapy goals in terms of frequency was as follows: a) symptom- and problem-related, b) personal growth-related, c) interpersonal-related, d) well-being-related, and e) existential-related therapy goals. For about half of the 645 patients, the set goals were selected from three distinct categories (*n* = 314, 48.7%), for about a quarter of the patients from four distinct categories (*n* = 168, 26.0%), for about one fifth of the patients from two distinct categories (*n* = 119, 18.5%), and for a vast minority from either one category (*n* = 25, 3.9%) or the maximum of five categories (*n* = 19, 2.9%).

**Table 1:** Descriptive Statistics

Variable	Goal selection		Goal attainment	
	Selected (at least) once	Never selected	*M* (range -3 to +3)	*SD*
Symptom related	620	25	1.96	0.88
Interpersonal related	387	258	1.85	0.99
Personal growth-related	434	211	1.90	0.84
Existential related	170	475	1.82	1.06
Well-being related	230	415	1.84	0.97

*Note*. *n* = 645; *M* = mean; *SD* = standard deviation.

The mean scores of the SCL-90R at the beginning (t_1_) and end (t_2_) of therapy for GSI t_1_ = 1.00 (*SD* = 0.57) and t_2_ = 0.48 (*SD* = 0.46). For the OQ-30, the mean scores before and after therapy were t_1_ = 1.69 (*SD* = 0.55) and t_2_ = 0.93 (*SD* = 0.56) for the overall score. The overall mean patients’ treatment satisfaction was 3.76 (*SD* = 0.32). A correlation matrix of the study variables, including the outcome prediction, is provided in Table 2.

**Table 2:** Correlation Coefficients Among all Study Variables

Variable	1	2	3	4	5	6	7	8	9
1. GSI pre	–								
2. GSI post	.496*	–							
3. OQ-30 pre	.779*	.408*	–						
4. OQ-30 post	.415*	.841*	.456*	–					
5. CSQ-8 post	-.082*	-.303*	-.090*	-.348*	–				
6. GAS symptom	-.077	-.448*	-.124*	-.532*	.342*	–			
7. GAS interpersonal	-.055	-.325*	-.123*	-.386*	.328*	.436*	–		
8. GAS personal growth	-.083	-.338*	-.139*	-.466*	.256*	.563*	.464*	–	
9. GAS existential	-.050	-.310*	-.049	-.358*	.344*	.588*	.578*	.461*	–
10. GAS well-being	-.084	-.344*	-.135*	-.394*	.181*	.497*	.504*	.466*	.548*

*Note*. Pre refers to the pre-treatment score, post to the post-treatment score; GSI = Global Severity Index; OQ-30 = Outcome Questionnaire; CSQ-8 = Client Satisfaction Questionnaire; GAS = Goal Attainment Scaling at the end of treatment.

*p < .05.

### Goal Selection

Table 3 presents the results of all the models. The symptom goal selection model showed an ICC of 0.428 in the null model, indicating substantial therapist-level variance on symptom goal selection. In the full model, the only significant predictor was interpersonal problems (*b* = -.705, *SE* = 0.355, 95% CI [-1.401, -0.010], *p* = .046), with greater interpersonal problems being associated with a decreased likelihood of selecting symptom goals (*OR* = 0.494). The ICC for the full model was 0.398, indicating that therapist differences continued to account for a substantial portion of the variance even after adjusting for patient characteristics.

**Table 3:** Predictors of Goal Type Selection

Baseline Characteristic	Goal categories					Goal diversity
	Symptom *Log-odds (SE)* *[95% CI]*	Interpersonal *Log-odds (SE)* *[95% CI]*	Personal growth *Log-odds (SE)* *[95% CI]*	Existential *Log-odds (SE)* *[95% CI]*	Well-being *Log-odds (SE)* *[95% CI]*	*Log-odds (SE)* *[95% CI]*
Sociodemographic						
Age	0.013 (0.021) [-0.029, 0.058]	0.005 (0.007) [-0.008, 0.019]	-0.003 (0.008) [-0.018, 0.012]	0.006 (0.008) [-0.009, 0.022]	0.004 (0.007) [-0.010, 0.018]	0.018* (0.007) [0.005, 0.031]
Gender (female)	-0.843 (0.604) [-2.026, 0.340]	0.218 (0.190) [-0.154, 0.589]	-0.017 (0.210) [-0.428, 0.394]	0.287 (0.215) [-0.134, 0.708]	-0.193 (0.191) [-0.568, 0.182]	0.195 (0.180) [-0.159, 0.548]
Symptom Checklist-90R – Subscales						
Somatization	0.114 (0.395) [-0.660, 0.888]	0.221 (0.129) [-0.032, 0.474]	-0.279* (0.137) [-0.547, -0.010]	-0.114 (0.138) [-0.385, 0.157]	-0.008 (0.127) [-0.256, 0.240]	-0.159 (0.121) [-0.395, 0.078]
Obsessive compulsion	0.265 (0.434) [-0.586, 1.115]	-0.135 (0.150) [-0.429, 0.159]	-0.046 (0.163) [-0.364, 0.273]	-0.239 (0.167) [-0.567, 0.089]	0.046 (0.150) [-0.248, 0.341]	-0.161 (0.138) [-0.431, 0.110]
Interpersonal sensitivity	0.057 (0.543) [-1.007, 1.121]	0.262 (0.180) [-0.089, 0.614]	-0.058 (0.192) [-0.434, 0.319]	-0.021 (0.193) [-0.398, 0.357]	-0.128 (0.179) [-0.478, 0.223]	0.069 (0.165) [-0.254, 0.392]
Depression	0.043 (0.518) [-0.972, 1.058]	0.238 (0.186) [-0.126, 0.602]	0.214 (0.199) [-0.175, 0.604]	0.448* (0.199) [0.058, 0.838]	-0.206 (0.186) [-0.571, 0.160]	0.371* (0.172) [0.034, 0.708]
Anxiety	-0.082 (0.462) [-0.987, 0.824]	-0.312* (0.154) [-0.613, -0.011]	0.112 (0.164) [-0.210, 0.434]	-0.091 (0.166) [-0.416, 0.234]	-0.089 (0.152) [-0.386, 0.208]	-0.144 (0.142) [-0.422, 0.135]
Hostility	-0.164 (0.340) [-0.830, 0.501]	-0.408* (0.134) [-0.671, -0.146]	0.105 (0.143) [-0.176, 0.386]	-0.125 (0.144) [-0.409, 0.158]	0.042 (0.133) [-0.220, 0.303]	-0.095 (0.121) [-0.332, 0.143]
Phobic anxiety	0.720 (0.503) [-0.266, 1.705]	-0.138 (0.120) [-0.374, 0.098]	-0.159 (0.129) [-0.412, 0.094]	-0.054 (0.134) [-0.318, 0.209]	0.084 (0.120) [-0.151, 0.320]	-0.084 (0.110) [-0.300, 0.133]
Paranoid ideation	0.083 (0.444) [-0.788, 0.953]	0.381* (0.164) [0.059, 0.703]	-0.002 (0.172) [-0.339, 0.335]	0.022 (0.172) [-0.315, 0.360]	0.134 (0.159) [-0.178, 0.447]	0.124 (0.149) [-0.168, 0.416]
Psychoticism	0.061 (0.439) [-0.799, 0.920]	0.120 (0.141) [-0.156, 0.395]	-0.027 (0.150) [-0.320, 0.267]	0.142 (0.150) [-0.153, 0.437]	0.012 (0.139) [-0.261, 0.284]	0.174 (0.128) [-0.076, 0.424]
Outcome Questionnaire-30 – Subscales						
Well-being	0.257 (0.519) [-0.760, 1.273]	-0.211 (0.187) [-0.578, 0.156]	-0.120 (0.202) [-0.516, 0.276]	0.053 (0.206) [-0.351, 0.457]	0.404* (0.188) [0.034, 0.773]	0.029 (0.173) [-0.310, 0.368]
Social role performance	-0.019 (0.369) [-0.743, 0.705]	-0.035 (0.135) [-0.298, 0.229]	0.036 (0.148) [-0.253, 0.326]	0.094 (0.147) [-0.196, 0.383]	-0.251 (0.135) [-0.516, 0.014]	-0.041 (0.126) [-0.288, 0.206]
Interpersonal problems	-0.705* (0.355) [-1.401, -0.010]	0.347* (0.125) [0.102, 0.593]	-0.016 (0.134) [-0.279, 0.247]	-0.118 (0.135) [-0.382, 0.147]	-0.213 (0.126) [-0.459, 0.033]	-0.098 (0.115) [-0.322, 0.127]
**Therapist-level variance (ICC)**	0.398	0.000	0.154	0.056	0.034	0.204

*Note*. ICC = intraclass coefficient; values represent log-odds coefficients from mixed-effects logistic (goal selection) and ordinal (goal diversity) regression models, with 95% Wald confidence intervals [CI] in brackets; overall scores from the SCL-90R (GSI) and OQ-30 were not included in the models due to multicollinearity.

*p < .05.

The interpersonal goal selection model showed an ICC of ~ 0.000 in the null model, indicating minimal therapist influence on interpersonal goal selection. Given the negligible therapist-level variance and boundary fit in the multilevel model, a sensitivity analysis was conducted using a single-level logistic regression. The results were identical in terms of fixed effect estimates and significance levels. In the full model, significant predictors included anxiety (*b* = -.312, *SE* = 0.154, 95% CI [-0.613, -0.011], *p* = .042, *OR* = 0.732) and hostility (*b* = -.408, *SE* = 0.134, 95% CI [-0.671, -0.146], *p* = .002, *OR* = 0.665), both of which decreased the likelihood of selecting interpersonal goals, as well as paranoid ideation (*b* = .381, *SE* = 0.164, 95% CI [0.059, 0.703], *p* = .020, *OR* = 1.464) and interpersonal problems (*b* = .347, *SE* = 0.125, 95% CI [0.102, 0.593], *p* = .006, *OR* = 1.415), which increased the likelihood. The ICC for the full model remained 0.000.

The personal growth-related goals model showed an ICC of 0.126 in the null model. In the full model, the only significant predictor was somatic symptoms (*b* = -.279, *SE* = 0.137, 95% CI [-0.547, -0.010], *p* = .042, *OR* = 0.757), which decreased the likelihood of selecting personal growth-related goals. The ICC for the full model was 0.154.

The existential goal selection model showed an ICC of 0.047 in the null model. The only significant predictor was symptoms of depression (*b* = .448, *SE* = 0.199, 95% CI [0.058, 0.838], *p* = .024, *OR* = 1.566), which increased the likelihood of selecting existential goals. The ICC for the full model was 0.056.

The well-being goal selection model showed an ICC of 0.030 in the null model. The only significant predictor in the full model was the OQ-30 subscale of well-being (*b* = .404, *SE* = 0.188, 95% CI [0.034, 0.773], *p* = .032, *OR* = 1.497), which increased the likelihood of selecting well-being goals. The ICC for the full model remained 0.034.

The goal diversity model showed an ICC of 0.167 in the null model. In the full model, significant predictors included patient age (*b* = .018, *SE* = 0.007, 95% CI [0.005, 0.031], *p* = .007, *OR* = 1.018), with older patients more likely to set more diverse goals, and depression (*b* = .371, *SE* = 0.172, 95% CI [0.034, 0.708], *p* = .031, *OR* = 1.449), which also increased the diversity of goals. The ICC for the full model was 0.204.

### Goal Selection and Attainment, and Their Association With Psychotherapy Outcomes

Building on the goal selection results, the second analysis examined whether the conditional attainment (as an interaction with the selection) of different therapy goals can predict residualized post-treatment scores in symptoms, global functioning, and end-of-treatment patient satisfaction (see Table 4).

**Table 4:** Predictors of Residualized Post-Treatment Scores in Symptoms, Global Functioning, and End-Of-Treatment Patient Satisfaction

Variable	Symptoms^a^	Global Functioning^b^	Patient Satisfaction^c^
	*PM (PSD)* *[95% CrInt]*	*PM (PSD)* *[95% CrInt]*	*PM (PSD)* *[95% CrInt]*
**Intercept**	-0.034 (0.078) [-0.190, 0.116]	-0.089 (0.099) [-0.286, 0.105]	29.735 (0.260) [29.288, 30.321]
Selection of goal at least once			
Symptom-related	0.308* (0.073) [0.167, 0.453]	0.537* (0.096) [0.352, 0.728]	-0.441* (0.246) [-0.999, -0.039]
Interpersonal-related	0.031 (0.043) [-0.053, 0.114]	0.038 (0.055) [-0.069, 0.146]	-0.090 (0.119) [-0.321, 0.148]
Personal growth-related	0.105* (0.046) [0.014, 0.195]	0.210* (0.062) [0.088, 0.331]	-0.151 (0.129) [-0.405, 0.106]
Existential-related	0.133* (0.053) [0.028, 0.236]	0.211* (0.070) [0.072, 0.347]	-0.134 (0.133) [-0.382, 0.143]
Well-being-related	0.118* (0.049) [0.020, 0.214]	0.059 (0.065) [-0.070, 0.187]	0.043 (0.131) [-0.194, 0.316]
Average goal attainment of:			
selection: symptom-related	-0.143* (0.016) [-0.175, -0.110]	-0.222* (0.023) [-0.267, -0.177]	0.112* (0.051) [0.014, 0.213]
selection: interpersonal-related	-0.039* (0.019) [-0.075, -0.003]	-0.040 (0.023) [-0.086, 0.006]	0.042 (0.055) [-0.063, 0.152]
selection: personal growth-related	-0.039* (0.020) [-0.078, -0.001]	-0.117* (0.026) [-0.169, -0.065]	0.062 (0.060) [-0.053, 0.182]
selection: existential-related	-0.031 (0.024) [-0.078, 0.017]	-0.058 (0.032) [-0.121, 0.006]	0.046 (0.063) [-0.076, 0.173]
selection: well-being-related	-0.060* (0.023) [-0.105, -0.015]	-0.038 (0.030) [-0.096, 0.020]	-0.017 (0.060) [-0.138, 0.098]
**Therapist-level variance (ICC)**	0.047	0.019	0.000

*Note*. PM = posterior mean; PSD = posterior standard deviation; CrInt = credible interval; ICC = intraclass coefficient.

^a^*n* = 636. ^b^*n* = 633. ^c^*n* = 627.

*p < .05.

For symptom change as the psychotherapy outcome, the mean attainment of symptom-, interpersonal-, personal growth-, and well-being-related goals was associated with greater reductions in symptoms, beyond what would be expected based on patients' initial severity. The ICC for symptom changes was 0.047, indicating that approximately 5% of the variance in symptom improvement was attributable to therapist differences.

For improvements in overall global functioning, higher mean attainment of symptom- and personal growth-related goals was associated with better-than-expected outcomes. The ICC for global functioning was 0.019.

For patients’ treatment satisfaction, only a higher mean attainment of symptom-related goals was associated with higher treatment satisfaction. The ICC for patient satisfaction was 0.000, suggesting that satisfaction was driven almost entirely by patient-level factors.

## Discussion

This study examined how different goal contents (categorized according to the BIT-C) of collaboratively defined therapy goals relate to psychotherapy outcome in outpatients receiving cognitive-behavioral psychotherapy, addressing two gaps in the current literature: which patient characteristics are associated with the selection of specific goal categories, and whether the attainment of different goal categories shows differential associations with treatment outcomes. The present findings show that therapy goal selection varies systematically with certain patient characteristics and partially with therapist factors, and that goal attainment is associated with better-than-expected treatment outcomes.

Descriptively, most patients selected symptom-related goals at least once, followed by personal growth and interpersonal goals, while existential and well-being goals were chosen less frequently. Attainment of symptom-related goals was generally higher than that of other goal types, with existential and interpersonal goals showing slightly lower levels of attainment. To contextualize, this pattern is largely consistent with prior studies conducted in CBT settings, where symptom-focused goals tend to dominate (Baur et al., 2024; Schöttke et al., 2014). It likely reflects the structured, problem-focused orientation of CBT, which emphasizes symptom reduction and functional improvement (Margraf & Schneider, 2009).

This interpretation is further supported by the goal selection results, which showed substantial therapist-level effects, particularly for symptom-related goals (≈ 40%), but also for goal diversity (≈ 20%) and personal growth–related goals (≈ 15%). When placed in perspective, these therapist effects appear substantial. Therapist effects in psychotherapy typically account for about 4–17% of outcome variance (see Wampold & Owen, 2021), about 6–13% of dropout variance (Saxon et al., 2017; Zimmermann et al., 2017), and roughly 11–16% of the variance in the alliance–outcome association (Del Re et al., 2012). Therefore, before discussing the potential predictor of goal selection, it is important to note that goal selection is (at least for some goal categories) strongly shaped by therapist guidance and treatment context, not only by patient characteristics. Nevertheless, several patient intake characteristics showed meaningful associations with goal content. For example, higher levels of interpersonal problems were associated with a lower likelihood of selecting at least one symptom-related goal and a higher likelihood of selecting at least one interpersonal-related goal. This suggests that patients tend to formulate goals in the domain where they experience high distress. In contrast, symptoms of depression were linked to a higher likelihood of selecting existential-related goals and greater goal diversity, the latter being consistent with prior findings (Grosse Holtforth et al., 2009). Interestingly, being older is also associated with a higher probability of selecting a more diverse set of therapy goals, potentially due to the addition of age-related challenges (Choi et al., 2021; Raue et al., 2017; Vathke et al., 2023). On the other hand, other symptoms showed a more selective shift of goal content rather than broader effects. For example, higher somatization was associated with a lower likelihood of selecting personal growth–related goals, which may reflect a stronger focus on bodily distress (Miller, 2008) and potentially a reduced capability for self-exploratory change processes (Ballespí et al., 2019; Mattila et al., 2008). Together, these findings indicate that therapy goal categories capture clinically meaningful differences in how patients conceptualize their difficulties at treatment entry. As such, structured goal categorization may serve as a useful bridge between individualized goal setting and more standardized approaches to case formulation and treatment planning.

Alongside the question of which therapy goal categories patients select, the present findings also illustrate how progress toward these goals (in the specific context of this study) relates to therapeutic change. Goal attainment represents a direct indicator of patient-perceived improvements with respect to defined change targets (Lloyd et al., 2019) and is a complementary perspective on treatment success beyond standardized symptom measures (Lindhiem et al., 2016). Across different domains of treatment success, from symptom distress to broader functioning and patient satisfaction, attainment within several goal categories (conditional on them being selected) was associated with greater-than-expected improvement relative to baseline severity, indicating that progress on individualized goals is meaningfully reflected in standardized outcomes. The strength of these associations differed across outcome domains. In this sample, symptom change showed the most consistent links with specific goal attainment, whereas associations with global functioning and patient satisfaction were fewer and more selective. One likely explanation lies in the CBT treatment context, which, as mentioned above, emphasizes symptom reduction. On the other hand, broader aspects of global functioning (e.g., interpersonal problems and role domains) may change more slowly and not be fully captured at post-assessment (e.g., Quilty et al., 2013). Importantly, some goal content categories were selected less frequently, which reduces statistical power for their attainment effects and cautions against interpreting non-significant associations as evidence of irrelevance. The main effects of goal category selection should be interpreted with particular caution as well. These coefficients should not be interpreted as indicating that selecting such goals leads to poorer outcomes. Rather, they reflect differences between patients who selected a given goal category and those who did not. Patients selecting certain goal types may present with more complex or burdensome problem profiles, which are generally associated with less favorable outcomes (e.g., Boswell et al., 2012).

What, then, should be made of these findings? They align with the view that psychotherapy outcome cannot be reduced to symptom change alone but encompasses multiple domains of personally meaningful improvement. Patient-reported outcome research has repeatedly demonstrated that patients define their therapy success in diverse ways. For example, a qualitative meta-synthesis by Chevance et al. (2020) identified a large number of distinct outcome domains that patients consider relevant. At a more integrative level, Zavlis et al. (2025) proposed that valued therapy outcomes cluster around three broad themes (love, meaning, and work; with the latter encompassing symptom reduction and functioning). Across these perspectives, a common conclusion emerges: standardized symptom measures capture only part of what patients experience as improvement. Accordingly, De Smet et al. (2020) suggest that changes in standardized outcome measures should be contextualized within patients’ subjective narrative. Against this background, the present results add a quantitative perspective by showing that attainment in specific patient-defined goal content domains is reflected in standardized outcome indicators, but not uniformly. Therefore, the bridge between individualized goals and standardized outcomes seems domain-sensitive and not generic. Clinically, the present findings suggest that domain-specific goal attainment ratings could complement the standardized outcome evaluation. At the same time, standardized intake measures may help identify dominant distress domains and thereby inform the collaborative formulation of therapy goals.

Several questions remain open for future research. First, goal selection as well as goal attainment–outcome associations may differ across therapeutic orientations and treatment settings (Schöttke et al., 2014). Second, it would be valuable to examine whether similar domain-specific patterns emerge when using other goal-based or idiographic outcome approaches (Cooper & Xu, 2023; Sales, Faísca, et al., 2023), and how the BIT goal domains map onto broader patient-defined outcome domains described in qualitative and mixed-methods research (Chevance et al., 2020; Zavlis et al., 2025). Future research should also continue to examine how different goal-domain frameworks align with patients’ own conceptualizations of change and outcome. Third, future studies could extend this work by examining associations between goal attainment and negative outcomes, such as deterioration or dissatisfaction with treatment (Steinbrenner et al., 2025). Finally, therapy goals were treated here as fixed at baseline, although goal priorities and meanings may shift over the course of treatment. Capturing the dynamic development of goals and their attainment over time may provide a more process-sensitive understanding of how individualized goals relate to therapeutic change.

### Limitations

Several limitations should be noted when interpreting the present findings. First, as mentioned above, the number of patients selecting certain goal categories, particularly existential and well-being-related goals, was relatively low, which may have reduced statistical power to detect meaningful effects in these domains. However, this lower frequency may also reflect other possibilities, such as lower perceived relevance, greater difficulty in articulating these goals, or differences in how they are framed during goal setting. Second, because only patients who reported at least three therapy goals were included a priori, the findings may generalize primarily to patients who are able and willing to engage in collaborative goal setting. As goal articulation is likely associated with motivation and therapeutic alliance (e.g., DeFife & Hilsenroth, 2011; Ryan et al., 2011), a higher number of reported goals (independent of their content or diversity) may itself reflect more favorable psychotherapy process characteristics. Third, all therapists in the study were CBT trainees, potentially limiting the generalizability of the results to other therapeutic orientations or more experienced clinicians. Fourth, goal types were categorized based on their position within the BIT structure. However, some goals may have conceptually overlapped across categories, introducing ambiguity in the classification. Finally, all outcome measures were based on patient self-report, where therapist-rated improvements could also be important indicators.

### Conclusion

This study highlights the clinical value of collaboratively defined therapy goals and their attainment as meaningful indicators of therapeutic outcome. Progress toward individualized goals was systematically reflected in standardized outcome measures, although not uniformly across goal content domains. Substantial therapist-level effects in goal selection indicate that goal formulation is shaped not only by patient characteristics but also by therapeutic context. Taken together, these results support the integration of individualized therapy goals alongside standardized measures to enable a more nuanced and patient-centered assessment of psychotherapy outcomes.

## Data Availability

The datasets generated and analyzed in the current study contain sensitive patient information and therefore cannot be shared publicly. Data may be made available upon reasonable request to the corresponding author, in accordance with institutional and ethical guidelines.

## Related Versions

We note that the data stem from the same outpatient clinic as in Schöttke et al. (2014), but the samples do not overlap, as the earlier study included patients treated between 2002 and 2010, whereas the present study draws on patients treated from 2014 onwards.

## Reporting Guidelines

This article is written according to the JARS-QUANT guidelines.

## Preregistration

The analyses for this study were not preregistered.
